# Digital Technologies to Enhance Infectious Disease Surveillance in Tanzania: A Scoping Review

**DOI:** 10.3390/healthcare11040470

**Published:** 2023-02-06

**Authors:** Ummul-khair Mustafa, Katharina Sophia Kreppel, Johanna Brinkel, Elingarami Sauli

**Affiliations:** 1 School of Life Sciences and Bioengineering, Nelson Mandela African Institution of Science and Technology, Arusha P.O. Box 447, Tanzania; 2 Department of Biological Sciences, Dar es Salaam University College of Education, Dar es Salaam P.O. Box 2329, Tanzania; 3 Department of Public Health, Institute of Tropical Medicine Antwerpen, Kronenburgstraat 43, 2000 Antwerpen, Belgium; 4 Department of Infectious Disease Epidemiology, Bernhard Nocht lnstitute for Tropical Medicine, Bernhard-Nocht-Straße 74, 20359 Hamburg, Germany; 5 German Center for Infection Research (DZIF), 38124 Hamburg, Germany

**Keywords:** infectious diseases, surveillance, health management information system, digital technologies, disease surveillance and response system

## Abstract

Mobile phones and computer-based applications can speed up disease outbreak detection and control. Hence, it is not surprising that stakeholders in the health sector are becoming more interested in funding these technologies in Tanzania, Africa, where outbreaks occur frequently. The objective of this situational review is, therefore, to summarize available literature on the application of mobile phones and computer-based technologies for infectious disease surveillance in Tanzania and to inform on existing gaps. Four databases were searched—Cumulative Index to Nursing and Allied Health Literature (CINAHL), Excerpta Medica Database (Embase), PubMed, and Scopus—yielding a total of 145 publications. In addition, 26 publications were obtained from the Google search engine. Inclusion and exclusion criteria were met by 35 papers: they described mobile phone-based and computer-based systems designed for infectious disease surveillance in Tanzania, were published in English between 2012 and 2022, and had full texts that could be read online. The publications discussed 13 technologies, of which 8 were for community-based surveillance, 2 were for facility-based surveillance, and 3 combined both forms of surveillance. Most of them were designed for reporting purposes and lacked interoperability features. While undoubtedly useful, the stand-alone character limits their impact on public health surveillance.

## 1. Introduction

Infectious diseases disproportionately affect populations in sub-Saharan Africa and are the leading cause of death in most low-and middle-income countries [1]. In recent years, the world has faced several diseases of epidemic potential, such as Ebola, the novel SARS-CoV-2, Mpox, and arboviruses [2,3,4]. Alongside morbidity and mortality, outbreaks strain already limited health system resources [5], accelerate violence against women and minority groups [6], and cause economic and political instabilities, as well as collapse of societies [7,8]. The WHO recommends conducting infectious disease surveillance in all counties to minimize the impact of outbreaks within and across national borders [9]. 

According to the WHO, an ideal infectious disease surveillance process in African countries is conducted through an integrated disease surveillance and response (IDSR) framework launched in 2010 [9]. According to this framework, infectious disease surveillance activities can be conducted in multiple ways [9,10,11]: (1) Health facility-based surveillance (HFBS), where data are obtained from patients visiting the health facilities. (2) Community-based surveillance (CBS), where data are provided by community representatives, including teachers, pharmacists, and community health workers (CHWs). (3) Active surveillance, where surveillance officers search for the data at the health facilities and/or in community settings. (4)Passive surveillance, where health officers or community representatives provide data to the top authorities as part of their routine responsibilities [11]. An ideal infectious disease surveillance process also involves eight core functions: case identification, reporting, investigation and confirmation, analysis and interpretation of data, preparedness and response actions, feedback provision, monitoring, and evaluation [9,10]. 

To accelerate outbreak detection and response in African countries, the WHO recommends integration of information and communication technologies, specifically mobile phones and computers, in the implementation of IDSR activities [9]. Globally, the integration of mobile phone-based and computer-based systems in infectious disease surveillance has shown significant gains [12,13,14]. The expansion of network and internet infrastructures in emerging nations such as Tanzania promises even more benefits [15]. 

In Tanzania, the use of mobile phone-based and computer-based systems to strengthen the health system has a long history [16]. From 1999 to 2019, more than 160 information and communication technologies based on mobile phones or computers were implemented in the country [16]. In terms of surveillance, the first national mobile phone-based surveillance system, named the electronic Integrated Disease Surveillance and Response (eIDSR)system, was implemented in 2015 and aimed at strengthening infectious disease surveillance activities throughout Tanzania [16]. Despite these initiatives, the country still faces untimely detection and response to infectious disease outbreaks. For example, since 1974, Tanzania has continuously reported cholera outbreaks every year, killing up to 13,078 people in 2018 [17]. Viral hemorrhagic fever outbreaks, such as severe dengue, occur repeatedly in the country, affecting 6917 people and causing 13 deaths in 2019 [18]. Recently (July 2022), an outbreak of a mysterious disease, confirmed to be leptospirosis, affected 20 people, causing 3 deaths [19]. 

Epidemiological surveillance for infectious diseases in sub-Saharan Africa (ESIDA) is a recently launched project that seeks to build an electronic health system to promote early identification and alert of epidemics at the local level [20]. The authors of this work, who are also project team members, conducted a literature review to compile information on the use of mobile phone-based and computer-based systems for infectious disease surveillance in Tanzania. In order to identify gaps, the ESIDA project should address and inform a future research agenda. This review aims to answer the question, “What mobile phone-based systems and computer-based systems have been developed and implemented for infectious diseases surveillance purposes in Tanzania?”Results from this review will provide information on the current situation in the country regarding the implementation and integration of mobile phones and computer-based technologies in the infectious disease surveillance process [21]. The PRISMA guidelines for scoping reviews were followed herein [22]. 

## 2. Materials and Methods

This review has taken into consideration the five steps recommended for scoping reviews [23,24]. These include identification of the research question, search for relevant studies, and selection of the most relevant studies applying inclusion and exclusion criteria, charting the data, and finally summarizing and reporting the results. 

### 2.1. Research Question

What mobile phone-based systems and computer-based systems have been developed and implemented for infectious disease surveillance purposes in Tanzania?

### 2.2. Information Sources and Search Strategy

Peer-reviewed articles were searched from the databases Cumulative Index to Nursing and Allied Health Literature (CINHAL), Excerpta Medica Database (Embase), PubMed, and Scopus. Non-peer-reviewed publications (gray literature) were searched using the worldwide Google search engine. The main reason behind the use of the general-purpose Google search engine to locate gray literature was based on the interest in capturing both scholarly and non-scholarly gray literature sources. Non-scholarly literature sources such as blog news, newsletters, reports, guidelines, and strategic plan documents from government and non-academic institutions were likely to be absent from scholarly sources such as Google Scholar. Despite its limitations [25], the Google search engine is applauded for its capacity to retrieve comprehensive lists of documents, thus increasing the scope of gray literature [25,26]. Search engines such as Google Scholar, in this case, would lead to duplication of efforts, as many of the scholarly articles it indexes [27] would have already been captured in the database search. The missing gray literature, however, is located through the general Google search engine.

A search query was developed by combining key terms used to document the integration of digital technologies in health and terms representing infectious disease surveillance. The most commonly used concepts in the literature to describe the integration of computers and mobile devices in the health sector are eHealth, mHealth, telehealth, and telemedicine [28]. eHealth refers to the application of information and communication technologies in the health sector [28]. mHealth is the use of mobile devices, mainly mobile phones, for health purposes [28]. Telehealth is the use of mobile technologies to connect health professionals and their clients [28]. Telemedicine describes the usage of mobile devices by health professionals in consultations to solve patients problems [28]. Several other concepts are included in specific mHealth-related literature, such as short message service (SMS), smartphone/mobile applications (mobile apps), and interactive voice response (IVR/VRS) [12]. For infectious diseases, the main terminologies used are surveillance and public health surveillance [12]. Combined with Boolean operators, the following search query was used for database search: (Telemedicine OR mHealth OR “mobile Health” OR Telehealth OR “electronic* Health” OR eHealth OR Mobile, Health OR “cell phone*” OR “cellular phone*” OR “mobile phone*” OR text* OR “Short Message Service” OR message* OR SMS OR “Interactive Voice Response” OR IVR OR VRS OR smartphone* OR “mobile application” OR “mobile app*” AND (Surveillance* OR “Pubic health surveillance”) AND (Tanzania OR “United Republic of Tanzania” OR Zanzibar) Filters: from 2012/1/1/to 2022/11/28). 

We adopted the search query from Brinkel et al. with slight modifications [12].The modifications were on geographic setting and time span. The previous study captured literature published before January 2014, while we captured literature published between the years 2012 and 2022. Keywords such as Africa and sub-Saharan Africa, used in the previous study, were replaced with the keywords Tanzania, United Republic of Tanzania, and Zanzibar.

The Google search was conducted using three search queries (“mHealth AND surveillance AND Tanzania,” “eHealth AND surveillance AND Tanzania,” and “Digital health AND surveillance AND Tanzania”).The three phrases were used independently in three steps. First search: “mHealth AND surveillance AND Tanzania,” second search: “eHealth AND surveillance AND Tanzania,” and third search:“Digital health AND surveillance AND Tanzania”).Use of phrases within quotation marks and Boolean operators for literature search is recommended [26,29]. 

Articles and gray literature published in the last 11 years (2012−2022) were covered as the most recent national guideline for IDSR implementation in Tanzania was revised in 2011 [30,31]. The first search was conducted in August 2022 and repeated on 28 November 2022. The lead author, Ummul-khair Mustafa (U.-k.M.), and supervisor Elingarami Sauli (E.S.) conducted the literature search independently. The results were compared and resolved for any disagreement.

### 2.3. Selection of Relevant Publications

All publications retrieved from the Google search engine and databases were imported to Rayyan software [32], and duplicates were removed using the software. The study selection process involved two phases. First, screening for title and abstracts and retention of publications that described two themes: mobile phone-based and computer-based systems and infectious disease surveillance activities in Tanzania. Second, applying inclusion and exclusion criteria to the identified studies and retention of publications that best answered the research question. The inclusion and exclusion criteria applied were content, context, language, and timeframe and information access, as summarized in Table 1. Selection of relevant studies in both the title and abstract screening phase and the application of inclusion and exclusion criteria phase were independently conducted by U.-k.M. and E.S. and later compared and resolved in case of any disagreement.

### 2.4. Data Charting and Synthesis

A data charting form was prepared by the research team in advance. Then, U.-k.M. and E.S. conducted the data extraction process independently, compared the results, and resolved any disagreement. For each publication, the form was used to record title, type of publication, author names and their affiliation and dates, name of the mobile phone-based systems or computer-based systems reported, purpose of its design, delivery channel to end users, surveillance approach addressed by the system, interoperability aspect (linkage with existing information and communication technologies for infectious diseases surveillance in Tanzania), information about pilot testing and scale-up, infectious disease coverage (single versus multiple diseases), geographic scope of implementation (mainland Tanzania, Zanzibar, or both mainland and Zanzibar), IDSR activities supported by each system, and impact for surveillance. Descriptive statistics were conducted using Microsoft Excel. 

## 3. Results

### 3.1. Results of the Literature Search

**Figure 1:**
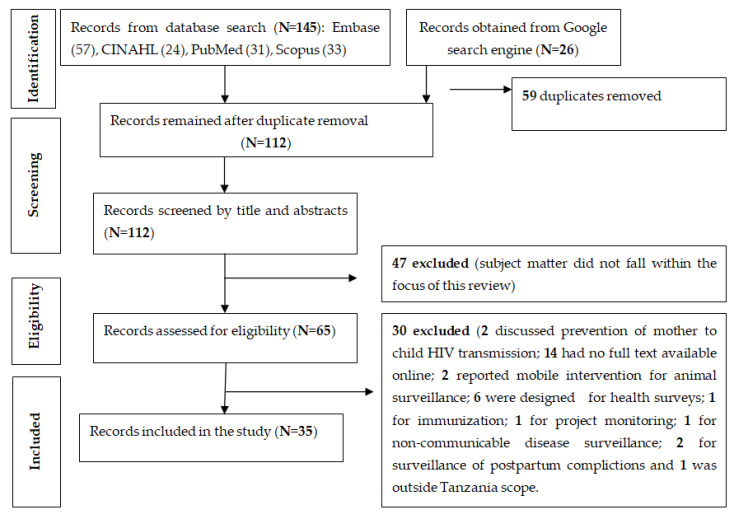
Flow diagram for publication selection process.

The searched databases yielded 145 publications: Embase (57), CINAHL (24), PubMed (31), and Scopus (33). After removing duplicates, screening for title and abstract, and assessing eligibility criteria, 9 documents remained. A literature search in the Google search engine yielded 26 relevant documents. In total, from all the searches, 35 publications were finally included in the review, as shown in Figure 1.

### 3.2. Characteristics of Publications Included in This Review

The publications included in this review were peer-reviewed papers (13, 37%), reports (6, 17%), manuals/guidelines (4, 11%), thesis/dissertation (4, 11%), strategic documents (3, 9%), program briefs (2, 6%), conference presentations (2, 6%), and blogs (1, 3%), as shown in Table 2. The majority of these publications were released in the year 2020 (7, 20%), followed by 2017 (6, 17%), 2016/2019 (5, 14.3% each), 2013 (4, 11.4%), 2018 (8.6%), and 2012/2021/2022 (1, 2.9% each), as shown in Table 2. Additionally, as indicated in Table 2, most of the publications were affiliated to academic/research institution (19, 54.2%), while the remaining were either published by the government of Tanzania/Zanzibar (22.9%) or non-academic institutions (22.9%), as shown in Table 2.

### 3.3. Characteristics and Benefits of Mobile Phone-Based Systems and Computer-Based Systems Implemented for Infectious Disease Surveillance in Tanzania

Thirteen digital technologies were identified in the review, 3 computer-based systems and 10 mobile phone-based systems accounting for 23.1% and 76.9% of total systems, respectively (Table 3). When all 13 systems were pooled and categorized by technology delivery channels, smartphone applications were the most commonly used technology (5, 39%), followed by web-based applications (3, 23%), and short-messaging service (SMS) systems (3, 23%), as shown in Table 3.

An interoperability feature was assessed for each of the reported systems; 7(54%) were stand-alone while the remaining 6(48%) were interoperable with existing national health management information systems/other electronic systems, addressing the infectious disease surveillance process in Tanzania, as shown in Table 4. 

The main IDSR activities supported by the identified systems were reporting (12, 92%), followed by data analysis (5, 39%), and a few technologies had detection/diagnosis and feedback functions (1, 8%), as shown in Table 4.

When the 13 systems were classified based on the surveillance approach, only 2(15%) were designed to assist CBS, 8(62%) aimed at HFBS alone, and the remaining 3(23%) were designed to address both CBS and HFBS, as shown in Table 4.

In terms of disease focus, 5(39%) the systems were designed to address surveillance of a single disease, mainly malaria and rabies. The remaining 8(62%) addressed multiple diseases, as shown in Table 4.

Furthermore, the 13 systems were classified according to geographic area of implementation; 8(62%) were designed to assist surveillance activities in mainland Tanzania only, 2(15%) in Zanzibar only, and 3(23%) in both mainland and Zanzibar, as shown in Table 4. 

Almost all reported digital surveillance systems were evaluated either during the testing and validation phase, pilot test, or rollout phase, and all were reported to be capable of achieving their target goal or objectives. Briefly, surveillance technologies contributed to four main areas: improvement of timeliness and completeness of infectious disease reports, increased number of reported events, detection of infectious diseases in community settings, and prevention of risks and costs associated with paper-based surveillance. The contributions or potential benefits of each reported technology are summarized in Table 5. 

### 3.4. Detailed Summary of Each Mobile Phone-Based System and Computer-Based System Identified in the Review

#### 3.4.1. Electronic Integrated Disease Surveillance and Response (eIDSR) System

eIDSR is a mobile phone-based system in the form of unstructured supplementary service data (USSD), which was developed in 2013 [33], for use at the health facilities to report immediate and weekly reportable infectious diseases in mainland Tanzania [10,34]. The electronic Integrated Disease Surveillance and Response (eIDSR)system was developed by the computer science and engineering department of the University of Dar es Salaam [35]. The eIDSR system was aimed at promoting rapid response to outbreaks and conditions of national and international concern by replacing paper-based submission of reports to higher authorities [35]. More precisely, surveillance officers at the facility level check the records for mandatory infectious diseases in the outpatient and inpatient registers on a weekly basis by summing the cases for each disease, recording the aggregate cases in the eIDSR booklet, and sharing the reports with top health authorities every Monday before 3:30 p.m. East African time [37]. The system was piloted in 2013 in four selected districts of Temeke (Dar es salaam region), Bunda (Mara region), Chato (Mwanza region), and Muleba (Kagera region) [37]. The eIDSR was then scaled up, reaching 100% coverage in 2020 [37]. The system is linked to the national health information system (HMIS) database DHIS2, and all information imported through eIDSR can be accessed by the top-level health authorities for further analysis and use [37,38]. Details of DHIS2 are provided in the following subsection. The implementation of eIDSR has been reported to contribute to improved timeliness and completeness of weekly reportable diseases above 80% of the national target [36,37].

#### 3.4.2. District Health Management Information System (DHIS2)

DHIS2 is a free, web-based application designed to assist data collection, management, analysis, and visualization by health authorities at different levels in order to guide their decision making [39,41]. With respect to infectious disease surveillance, DHIS2 is used for reporting the monthly summary of all diseases diagnosed at health facilities through the outpatient department (OPD) and inpatient department (IPD) modules [38,39]. DHIS2 system has its complementary paper-based health information system known as Mfumo waTaarifa za Uendeshaji Huduma za Afya (MTUHA), which is used in collecting individual patient records that are subsequently aggregated and recorded in monthly OPD/IPD summary forms [37]. Every month, the health facilities have to send the forms to the district MTUHA focal person for entry into DHIS2 [40,41]. Some health facilities with computers, electricity, and the internet can enter data directly to DHIS2 [40,41]. The national day for data entry in the DHIS2 system is every 15th date [37]. DHIS2 is linked to the eIDSR system described in the previous subsections [38,39]. Data analysis and visualization modules in DHIS2 are used to analyze and generate district, regional, and national reports, respectively [39]. After analysis of data uploaded through DHIS2, Tanzanian citizens and interested stakeholders can check reports through the national DHIS2 web portal available at https://hmisportal.moh.go.tz/ (accessed on 20 January 2023) [39].

DHIS2 was developed by the Health Information System Project (HISP) at the University of Oslo with support from other stakeholders: universities, ministries of health, as well as international health agencies [41]. Mainland Tanzania adopted DHIS2 in 2010 as a national HMIS database [41]. The pilot test for DHIS2 was conducted in the Pwani region, where national rollout was started in 2012 and completed in 2013 [41]. Similar to mainland Tanzania, Zanzibar also uses DHIS2 as an electronic HMIS database to capture all HMIS indicators [42]. 

#### 3.4.3. AfyaData App

AfyaData app is an Android smartphone application for supporting CBS of human, animal, and zoonotic diseases [43,44]. In Tanzania, registered CHWs use AfyaData to communicate data on health events captured during their routine visits to the households. Apart from data collection, AfyaData is equipped with one health knowledge repository database, which allows the app to predict, in percentage, the most likely diseases affecting the reported human or an animal. This component assists in confirmation of health events when there is no access to laboratory diagnosis [43,44]. The development of the AfyaData app began in 2010, and it was piloted in Ngorongoro and Morogoro Urban districts in Tanzania from August 2016 to December 2016 [43,44]. The AfyaData was scaled to other regions and has facilitated CBS in Kilosa, Malinyi, Ulanga, Ngara, Ngorongoro, and Wete districts of Tanzania [43].

The impact of AfyaData app includes facilitated reporting of community-based events (1816 animal cases and 99 human cases) during pilot, aid patient to visit to health facilities to receive proper diagnosis and treatment, supported timely detection and control of some epidemics, such as a mysterious disease of goats and sheep in Korombo, which was confirmed to be ovine rinderpest and contagious caprine pleuropneumonia (PPR/CCPP diseases), Impetigo-like outbreak in school children, East Coast fever infections in castles, rabies epidemic in the Ulanga, and anthrax in Ngorongoro [43,44]. 

#### 3.4.4. Mobile Phone-Based Surveillance System for Rabies

The mobile phone-based surveillance system for rabies is a mobile application in the form of SMS that was designed to facilitate the surveillance of rabies in southern Tanzania. The application is designed for reporting of animal bite cases, human rabies deaths, and vaccination records. The application was developed by a PhD student in 2010 in an effort to facilitate surveillance of rabies across the southern regions, which were implementing rabies control programs [46,47]. Pilot testing was conducted from 2011 to 2015 in Zanzibar island (Pemba) and three regions in mainland Tanzania: Morogoro (Ulanga and Morogoro rural), Pwani (Rufiji, Kibaha rural, and Kisarawe), Lindi (Nachingwea), and Mtwara (Masasi). Its impact in rabies surveillance includes increased number of reported cases (up to 29,595 cases were reported during pilot phase), increased timeliness of the reports (four times compared to paper-based system), and increased completeness of the reports (maximum of 100% compared to 96% for paper-based system) [46,47]. 

#### 3.4.5. Integrated Bite Case Management (IBCM) Application

Integrated bite case management (IBCM) application is an Android-based smartphone application developed to facilitate the implementation of an IBCM approach in Tanzania [48]. The aim of the IBCM is to facilitate the control of rabies by using a one-health approach, which requires collaboration between the public health sector and the veterinary sector [48]. The application has three main functionalities: data collection and reporting forms used to record animal bite cases at the health facilities, an epidemiological form for data collection and reporting of animal investigations in the field, and a high risk assessment functionality for notification of high-risk cases for triggering an animal investigation process [48]. The pilot phase of the IBCM approach was implemented from June 2018 to August 2019 in the four regions of Tanzania (Mtwara, Lindi, Mara, and Morogoro).The impacts of the IBCM application are: following its introduction in health facilities, there was a twofold increase in the number of reported bite victims and identification of high-risk victims; reported bite cases increased from an average of 55.7 to 92.2 cases, while detection of high-risk cases increased from 26.9% to 64.9% [48]. The IBCM application helped with the investigation of 823 suspected rabid animals, of which 404 animals were proven to bear signs or evidence of being infected with the rabies virus [48].

#### 3.4.6. Malaria Epidemic Early Detection System

The malaria epidemic early detection system (MEEDS) is a mobile application in the form of a USSD developed in 2008 for reporting of weekly malaria data at health facilities in Zanzibar [49,50,51]. It reports weekly aggregated data on outpatient visits, confirmed malaria-positive cases, and confirmed negative cases [51]. A notification component was added in 2012 to support district malaria surveillance officers in conducting active malaria surveillance (AMS) at the household [51]. The application has improved malaria reporting rates throughout Zanzibar. For example, malaria reporting rates increased from 69% to 86% for Unguja between July 2017 and July 2018 and from 58% to 93% for Pemba [52]. In addition, the system enables detection and response to malaria outbreaks within two weeks of onset by a surveillance team [50]. 

#### 3.4.7. Coconut Surveillance Application

Coconut Surveillance is a web-based application developed to facilitate AMS in Zanzibar [49,50,53,54].The application runs on tablets and is used for reporting malaria cases found at the household level [53,54]. National-level surveillance monitoring and evaluation team members use the inbuilt analysis functionality to analyze malaria data on a daily basis to detect districts/villages with abnormal malaria case numbers and consequently initiate malaria interventions [50]. Coconut Surveillance is linked to DHIS2 to enhance the availability of malaria data beyond national malaria control officers [55]. The application has contributed to malaria elimination efforts in Zanzibar. Within the six months of its implementation in public health facilities (July–December 2012), the application facilitated household follow-up for 980 cases and detection of 223 previously unknown malaria cases [56,57].

#### 3.4.8. Community-Based Disease Surveillance and Treatment of Malaria System (ComD-STM)

The community-based disease surveillance and treatment of malaria (ComD-STM) system is a smartphone Android-based mobile application developed for the surveillance of malaria, including treatment failure [58]. It was developed through collaboration of researchers from the National Institute for Medical Research (NIMR) in Tanga Tanzania and the University of Notre Dame, Indiana (USA) [58]. The ComD-STM was designed to facilitate community-based surveillance of malaria cases at household level but also captured data on patients attending health facilities at their own initiatives. It captured data on the patients’ clinical symptoms, laboratory diagnosis, treatment adherence, and treatment outcomes. The ComD-STM application was piloted in the Muheza district in the Tanga region of Tanzania between November 2013 and October 2014. During the pilot phase, the ComD-STM enabled identification of 1907 febrile patients through active and passive surveillance processes, reporting of 1778 patients to health facilities for malaria diagnosis and treatment and detection of 860 malaria cases by rapid test and 798 malaria patients by microscopy as well as detection of 9 malaria treatment failure [58]. 

#### 3.4.9. Smartphone-Based Reporting Application for Routine Health Data from Primary Health Facilities to the District Hospital

The smartphone-based reporting application is an Android-based application that was developed by a master’s student from Nelson Mandela African Institution of Science and Technology, Arusha Tanzania in 2016 [59,60]. The application aimed at replacing paper-based surveillance of weekly and monthly infectious diseases [59,60]. In paper-based surveillance, the health facilities submitted all the reports physically to the district medical officers [59]. With the smartphone application, weekly, and monthly reports would be recorded in digital form and transferred directly to the DHIS2 database through the internet [59,60]. The smartphone-based reporting application was tested in the Arumeru district in the Arusha region and the Kisarawe district in the coastal region of Tanzania [59]. During the testing phase, the application was associated with several benefits: contribution to timely reporting, reduction of costs incurred in the submission of reports, ease of the reporting process, reduction of risk of accidents during travel, especially during the rainy season, and provision of more time for health officers to complete other duties [59,60]. 

#### 3.4.10. Mobile Application for Collecting Integrated Disease Surveillance and Response Data

The mobile application for collecting integrated disease surveillance and response data is an SMS-based mobile application designed to support health workers in submitting weekly IDSR reports to the district level in Tanzania. The application was developed and tested by the Action research project in 2012 [61]. The development of the application was rooted in the DHIS2 mobile phone module, which allows entering the data into DHIS2 software by using mobile devices [61]. The application aimed at substituting paper-based reporting of weekly IDSR data, which had some shortcomings, including low completeness and timeliness of reporting as well as cost and time issues [61]. The mobile application was tested in the Kibaha town district and Kisarawe district in the Pwani region, Tanzania for a three-month period. It helped to improve the timeliness of the reports from 50% to 89%. The mobile application was also reported to have saved health workers time and travel expenses, which were huge compared to the cost incurred for sending an SMS [61].

#### 3.4.11. SMS and Smartphone Application for Disease Surveillance in Humans and Animals

A combination of a smartphone Android application and an SMS application were designed for capturing and transmitting disease event data in both the animal and the human sector [62]. The two digital technologies were founded by the Southern African Centre for Infectious Disease Surveillance (SACIDS) in 2010 [62]. Specifically, the two technologies aimed to assist the submission of community health reports for both human- and animal-related events, weekly and monthly reports for human diseases at health facilities, as well as animal surveillance by the veterinary sector [62].

The smartphone application was piloted in Tanzania (Ngorongoro and Ngara districts), Burundi (Muyinga district), and Zambia (Kazungula and Sesheke districts) from 2011 to 2013. During the pilot process, the smartphone technology facilitated submission of 1651 reporting forms and facilitated medical and veterinary offices in writing disease case reports, as well as conducting follow-upon the reported cases [62]. SMS technology was not used during the pilot phase; however, the technology demonstrated its capacity to fulfill the intended task when it was used for data collection in research activities in 2013 [62].

#### 3.4.12. Government of Tanzania—Hospital Management Information System (GoTHoMIS)

The GoTHoMIS is a web-based application designed to digitalize the various processes undertaken in the cycle of service provision at the health facilities [63]. In relation to infectious disease surveillance, GoTHoMIS version 3 has an environmental module that allows health officers to register notifiable diseases, and an outbreak sub-module to generate three types of reports (summary report, outbreak summary, and patient list) [63]. Moreover, the GoTHoMIS has a health management information system module known as MTUHA, which enables automatic generation of infectious disease monthly reports [66]. However, the data captured through the GoTHoMIS are stored on local savers at the respective health facilities, and the system is linked to neither the MTUHA module in DHIS2 nor the mobile phone eIDSR for weekly and immediate reportable diseases [38,64]. The system was piloted in Tumbi Hospital (Kibaha district) in 2012 and scaled up, gradually reaching more than 500 health facilities by 2020 [65]. Despite the presence of surveillance functionalities in the GoTHoMIS system, there are no data on its use for the surveillance process of infectious diseases.

## 4. Discussion

The objective of this review was to summarize the available literature on current and previous mobile phones and computer-based systems employed to facilitate infectious disease surveillance activities in Tanzania. 

The review found that efforts have been in place to incorporate information and communication technologies in the IDSR system in Tanzania. The mobile system eIDSR was reported to be used throughout mainland Tanzania for reporting of priority diseases at the health facilities, while a computer-based system DHIS2 was used nationwide at the district level for analysis and communication of mandatory IDSR reports to the top levels, community, and other stakeholders. This is similar to other African countries, including Kenya and Uganda, where eIDSR and DHIS2 have been adopted for the same purpose [67].

Mobile phone-based technologies such as MEED and Coconut Surveillance have made significant contributions toward eliminating malaria on Zanzibar Island. However, the malaria burden is still higher in mainland Tanzania, with more than 10regions recording a prevalence above 10% for children under five in 2017 [68]. Thus, expansion of the MEED and Coconut Surveillance in high-risk regions of the mainland could speed up the country’s attempts to fight malaria.

Most of the mobile phone- and computer-based interventions found in this review were implemented to facilitate only HFBS. Despite the fact that there is a significant potential for outbreak detection and response in health facilities, a tendency for late presentation to medical facilities, lack of access to medical facilities, and community members’ choice for alternative treatments all play a role in the slow diagnosis and response to outbreaks [69]. In addition to HFBS, CBS is strongly encouraged to reduce the effects of outbreaks [69]. Mobile phone applications, such as AfyaData identified in this review, have reported good impacts in accelerating outbreak detection and response through implementation of CBS platform. Its expansion to other areas is expected to have a greater impact on the entire nation. Studies from a number of nations, notably Kenya and Uganda, have shown how integrating CBS with digital technologies such as mobile applications can speed up the detection of epidemic prone diseases [69].

One of the shortcomings of some mobile phones and computer-based interventions for disease-specific programs was the lack of an interoperability feature. Interoperability is the term used to describe how well-established digital technologies can communicate with one another and how easily users can access and use data to make informed decisions [70]. Newly established electronic systems should be interoperable with the national health information systems, such as eIDSR and DHIS2, for the case of Tanzania [71]. System interoperability has a number of advantages, including improved service provider access to information, reduced workload, reduced data collection errors, improved coordination between healthcare sectors, maintenance of patient records, reduction of unnecessary costs, and improved data collection, analysis, and data access for research [70,72]. The GoTHoMIS application, which can automatically produce eIDSR and DHIS2 inputs, is noteworthy in this review. However, due to a lack of interoperability, the application is underutilized. 

Almost all mobile phones and computer-based interventions found in this review aimed at improving data management aspects, mainly submission of weekly, monthly, or disease-specific reports from the health facilities to the top levels. In addition to reports being sent late, the Tanzania IDSR system had other issues that needed to be addressed. For instance, past findings demonstrate that a lack of case definitions at the health facilities and a dearth of diagnostic tools and supplies make it difficult to correctly identify outbreaks [18,73]. To improve case detection, mobile phones and computer-based interventions integrating machine learning can assist physicians to conduct syndromic-based diagnosis [74]. Other areas that call for digital interventions are infectious disease screening and contact tracing, promoting public awareness and encourage vaccination habit, prediction and forecasting of outbreaks, as well as training of health workers and treatment of outbreak victims [74,75,76]. 

## 5. Limitations of the Review

Due to a number of factors, this evaluation may not have included all publications that discuss the use of digital technology for infectious disease surveillance in Tanzania. The search methodology used herein placed a special emphasis on infectious disease surveillance. This may have led to the absence of articles describing specific diseases without connecting them to the surveillance of infectious diseases. The search was only limited to works published between the years of 2012 and 2022; hence, some digital interventions that were created and implemented within the reporting period but were not published or indexed may not have been included in this review. This review only covered publications that were authored in English. This could result in critical government documents being overlooked that are written in Tanzania’s official language of Swahili. Because so many different publication categories were covered in this review, it was challenging to evaluate the quality of the published articles. However, to reduce this bias, a thorough search was carried out, which included looking through gray literature on the Google search engine using search phrases as described. Finally, multiple documents reporting were evaluated on the same digital intervention to increase the chances of learning everything about the herein reported tools. For instance, in addition to reading peer-reviewed articles that discussed a certain digital tool, theses, dissertations, or government and organization reports were also examined to see how they were connected to the implementation process to fill in any gaps in knowledge.

## 6. Conclusions

We conclude that progress has been made in the field of digital surveillance in Tanzania as we identified 13 digital technologies designed to aid in infectious disease surveillance activities between the years 2012 and 2022. Digital surveillance systems implemented in Tanzania utilized a variety of technologies, ranging from SMS, USSD, and smartphone applications, as well as web-based applications, and all of them demonstrated promising results. However, we found four major weaknesses: (1) the majority of the applications are aimed at facilitating HFBS, especially reporting of communicable infectious diseases through the routine IDSR reporting process, meaning there is little investment in community-based surveillance; (2) nearly half the technologies were found to lack interoperability features, thus limiting their impacts in surveillance; (3) there is fragmentation in implementation of digital surveillance technologies across mainland Tanzania and Zanzibar, indicating digital divide within the country; (4) initial steps in the surveillance activities are still paper-based, causing data discrepancy and hard work of data compilation for reporting process. Recommendations to the government and stakeholders for practice, as well as future research directions to bridge the identified gaps, are offered in the following subsections. 

### 6.1. Implications for Practice

Future development and research into the interoperability of surveillance digital technologies is highly recommended. Developed nations are in a better direction as their health systems are interoperable; thus, research on what they did and how best Tanzania can do it are required. Moreover, the Tanzanian government is encouraged to scale up the geographic scope of existing community-based digital interventions that have proven to be effective in pilot settings or small geographic settings (Coconut Surveillance and AfyaData app). As these interventions are supported by donors, a request for project expansion to new settings has to be made to funders. By contrast, the mainland Tanzania government can think of adding community-based functionalities available in Coconut Surveillance/AfyaData into existing nationally distributed technologies such as eIDSR. In the same view, Zanzibar can also benefit from a multiple disease’s community-based surveillance approach of AfyaData by incorporating AfyaData functionalities into Coconut Surveillance, which is currently in use for community-based malaria surveillance throughout Zanzibar.

A special recommendation is made toward ensuring full digitalization of surveillance activities at the health facilities. Expanding GoTHoMIS, which has demonstrated the capacity to fully digitalize the entire IDSR system from data collection to the reporting process, can achieve this. Adoption of such a system would require funds for purchase of computers, training of health professionals, monitoring and supervision, as well as system maintenance. To make this possible, the government of Tanzania can request support from donors or use the district and health facilities revenue. Similarly, to avoid cost and duplication of efforts, the government can consider expanding access to DHIS2 (already in use throughout the nation) to all health facilities and then incorporate the GoTHoMIS functionalities into the DHIS2 system to allow health workers to capture patient data needed for surveillance in electronic systems at the start of patient admission rather than going to tack patient records in paper forms at the end of a month or week. Last, we advocate for the development of digital surveillance technologies with multiple functionalities, especially the inclusion of decision support systems, education, and data analysis capacity.

### 6.2. Implications for Future Research

This review was limited to the identification of mobile phone-based systems and computer-based systems designed and implemented for infectious disease surveillance in Tanzania and did not examine the quality of reported publications. Future research should consider conducting a systematic review to examine the quality of evidence of publications on infectious disease surveillance in Tanzania. As half of the reported surveillance systems seem to lack an interoperability feature, future reviews may consider an evaluation of best practices to make digital surveillance systems interoperable. In addition, the subject of community-based surveillance is still uncovered in most of the reported evidence found in Tanzania; future review may consider an assessment of community-based surveillance technologies implemented in Africa and worldwide to inform Tanzania and other countries. Last, the reported digital technologies seem to have limited functionalities, as the majorities were designed to facilitate the reporting of data captured at health facilities or community settings. In areas such as Tanzania with limited laboratory capacity, health workers’ decision support algorithms would be a valuable additional tool in disease detection; thus, we recommend future reviews on decision support algorithms implemented for surveillance activities in Africa and worldwide to help Tanzania and other countries improve this aspect. 

## 7. Review Summary and Future ESIDA Project Directions

The ESIDA project conducted a scoping review to identify digital technologies implemented for infectious diseases surveillance activities in Tanzania between 2012 and 2022. The overall aim was to understand the work that has already been done in the field and to identify the gaps that ESIDA can fill. A PhD student with supervision support from project partners in Tanzania and Germany conducted the review. All recommended standard steps for conducting a scoping review were followed. Peer-reviewed articles were searched from the health databases PubMed, Embase, Scopus, and CINAHL, while an advanced general Google search engine was used to locate non-scholarly documents. The search was conducted between August and November 2022.Thirty-five publications reporting on digital technologies designed and implemented for infectious diseases surveillance in the country were obtained; interest variables (author, year of publication, document themes, and reported technology, its aim of design, implementation process, and outcomes) were summarized. 

From the review, we learned that progress has been made in Tanzania in integrating digital technologies such as computers and mobile phones in the infectious diseases’ surveillance processes. However, the following few gaps were also identified:

- The majority of the applications are aimed at facilitating HFBS, especially the reporting of communicable infectious diseases through the routine IDSR reporting process, meaning there is little investment in community-based surveillance.
- Furthermore, nearly half the technologies were found to lack interoperability features, thus limiting their impacts in surveillance.
- There is fragmentation in the implementation of digital surveillance technologies across mainland Tanzania and Zanzibar, indicating a digital divide within the country.
- Initial steps in the surveillance activities are still paper-based, causing data discrepancy and hard work of data compilation for the reporting process.

The next step in the ESIDA project is development of a mobile application decision support and surveillance system that shall focus on enabling health professionals in screening of notifiable infectious diseases outlined in the Tanzania IDSR guidelines as well as transmission of data to the ESIDA hub for further risk assessment incorporating epidemiological data, historical and environmental data, as well information from available open data sources to support an outbreak prediction and alert in real time. 

## Figures and Tables

**Table 1:** Inclusion and exclusion criteria.

Criterion	Inclusion	Exclusion
Content	Any publication on the theme of application of mobile phone-based and computer-based systems for infectious disease surveillance	Publications on non-communicable disease surveillance and publications on application of mobile phone-based and computer-based systems for treatment adherence, health education or disease survey
Context	Tanzania	Publication outside the scope of Tanzania
Language	English	Any publication in other languages
Timeframe	2012–2022	Any publication outside the set timeframe
Information access	Full text document	Any publication with no full text

**Table 2:** Summary of included publications.

Author (Affiliation)	Title	Year	Category
Ministry of Health and Social Welfare [33] (Government)	Tanzania national eHealth strategy, June 2013–July 2018.	2013	Strategic document
Maternal and Child Survival Program [34] (Non-academic institution)	Disease surveillance MCSP Tanzania program brief.	2019	Program brief
Nkowane [10] (Non-academic institution)	Streamlining and strengthening the disease surveillance system in Tanzania, disease surveillance system review, asset mapping, gap analysis, and proposal of strategies for streamlining and strengthening disease surveillance.	2019	Report
United Republic of Tanzania [35] (Government)	mHealth-eIDSR project status, September 2013.	2013	Report
Joachim [36] (Academic/research institution)	Electronic integrated disease surveillance and response eIDSR implementation in Tanzania.	2018	Conference presentation
Joseph [37] (Academic/research institution)	Improvements in malaria surveillance through the electronic Integrated Disease Surveillance and Response (eIDSR) system in mainland Tanzania, 2013–2021.	2022	Peer-reviewed article
Rutatola [38] (Academic/research institution)	A framework for timely and more informative epidemic diseases surveillance: The case of Tanzania.	2018	Peer-reviewed article
Ministry of Health, Community Development, Gender, Elderly, and Children [39] (Government)	DHIS2 functions and data use for health information system.	2017	Guideline
Ministry of Health, Community Development, Gender, Elderly, and Children [40] (Government)	Health Data Collaborative (HDC) Implementation Report.	2020	Report
Sukums [41] (Academic/research institution)	Avoiding pitfalls: Key insights and lessons learned from customizing and rolling out a national web-based system in Tanzania.	2021	Peer-reviewed article
Revolutionary government of Zanzibar [42] (Government)	Zanzibar digital health strategy, 2020/21-2024/25.	2020	Strategic document
Fondation Pierre Fabre [43] (Non-academic institution)	Field survey report AfyaData Project: Promoting proper management of zoonotic diseases through e-based one health training of frontline health worker.	2019	Report
Karimuribo [44] (Academic/research institution)	A smartphone app (AfyaData) for innovative one health disease surveillance from community to national levels in Africa: Intervention in disease surveillance.	2017	Peer-reviewed article
Mpolya [45] (Academic/research institution)	Toward elimination of dog-mediated human rabies: Experiences from implementing a large-scale demonstration project in southern Tanzania.	2017	Peer-reviewed article
Mtema [46] (Academic/research institution)	Integrated disease surveillance and response systems in resource–limited setting.	2013	Doctoral thesis
Mtema [47] (Academic/research institution)	Mobile phones as surveillance tools: Implementing and evaluating a large-scale intersectoral surveillance system for rabies in Tanzania.	2016	Peer-reviewed article
Lushasi [48] (Academic/research institution)	One health in practice: Using Integrated Bite Case Management to increase detection of rabid animals in Tanzania.	2020	Peer-reviewed article
Ministry of health, Zanzibar [49] (Government)	National guidelines for malaria surveillance and response.	2016	Guideline
Ministry of health, Zanzibar [50] (Government)	Malaria surveillance in Zanzibar: Data analysis and interpretation.	2016	Guideline
U.S. President’s Malaria Initiative Tanzania, Zanzibar [51] (Non-academic institution)	Malaria operational plan, FY 2020.	2020	Strategic document
MEASURE Evaluation-Tanzania [52] (Non-academic institution)	MEASURE Evaluation—Tanzania’s technical assistance for malaria surveillance in mainland Tanzania and Zanzibar: Progress, 2016–2018.	2018	Program brief
Van Der Horst [53] (Academic/research institution)	Operational coverage and timeliness of reactive case detection for malaria elimination in Zanzibar, Tanzania.	2020	Peer-reviewed article
Khandekar [54] (Academic/research institution)	Evaluating response time in Zanzibar’s malaria elimination case-based surveillance-response system.	2019	Peer-reviewed article
Vital Wave [55] (Non-academic institution)	Mobile solutions for malaria elimination surveillance systems: A roadmap.	2017	Report
Cressman [56] (Academic/research institution)	Using mobile technology to help eliminate malaria in Zanzibar.	2014	Conference presentation
Neat [57] (Academic/research institution)	Use of technology in malaria prevention and control activities.	2013	Blog
Francis [58] (Academic/research institution)	Deployment and use of mobile phone technology for real-time reporting of fever cases and malaria treatment failure in areas of declining malaria transmission in Muheza district north-eastern Tanzania.	2017	Peer-reviewed article
Pascoe [59] (Academic/research institution)	Electronic information capturing, processing, and reporting of routine health data using smartphone-based applications.	2016	Master’s dissertation
Pascoe & Mwangoka [60] (Academic/research institution)	A smartphone-based reporting application for routine health data: System requirements, analysis, and design.	2016	Peer-reviewed article
Pascoe [61] (Academic/research institution)	Collecting integrated disease surveillance and response data through mobile phones.	2012	Peer-reviewed article
Mwabukusi [62] (Non-academic institution)	Mobile technologies for disease surveillance in humans and animals.	2014	Peer-reviewed article
President’s Office—Regional Administration and Local Government [63] (Government)	Government of Tanzania—hospital management information system (GoT-HOMIS) version 3.0) User Manual v1.	2017	Manual
Rutatola [64] (Academic/research institution)	Monitoring spread of epidemic diseases by using clinical data from multiple hospitals: A data warehouse approach.	2020	Master’s dissertation
United States Agency International Development [65] (Non-academic institution)	Cost efficiency, revenues, and expenditure assessment of four public systems strengthening interventions in Tanzania.	2020	Report
Peltola [66] (Academic/research institution)	On adoption and use of hospital information systems in developing countries: Experiences of health care personnel and hospital management in Tanzania.	2019	Master’s thesis

**Table 3:** Types of digital technologies identified from the literature review.

Digital Surveillance Name	Technology Used	Delivery Channel
Electronic integrated disease surveillance and response system [10,33,34,35,36,37,38]	Mobile phone	Unstructured supplementary service data
Malaria epidemic early detection system [49,50,51,52]	Mobile phone	Unstructured supplementary service data
AfyaData app [43,44]	Mobile phone	Smartphone application
Integrated bite case management application [48]	Mobile phone	Smartphone application
Community-based disease surveillance and treatment of malaria system [58]	Mobile phone	Smartphone application
Smartphone-based reporting application [59,60]	Mobile phone	Smartphone application
Smartphone application for surveillance in humans and animals [62]	Mobile phone	Smartphone application
Mobile phone-based surveillance system for rabies [45,46,47]	Mobile phone	Short message service
SMS application for surveillance in humans and animals [62]	Mobile phone	Short message service
Mobile application for collecting integrated disease surveillance and response (IDSR) data [61]	Mobile phone	Short message service
District health management information system [38,39,40,41,42]	Computer	Web-based application
Coconut Surveillance [49,50,53,54,55,56,57]	Computer	Web-based application
Government of Tanzania—hospital management information system [63,64,65,66]	Computer	Web-based application

**Table 4:** Summary of characteristics of identified mobile phone-based systems and computer-based systems designed for infectious disease surveillance in Tanzania.

Variable	Technologies in the Category
Interoperability	eIDSR [10,33,34,35,36,37,38]; DHIS2 [38,39,40,41,42]; MEEDS [49,50,51,52]; Coconut Surveillance [49,50,53,54,55,56,57]; mobile application for collecting IDSR data [61] and smartphone-based reporting application [59,60].
No Interoperability	AfyaData app [43,44]; Mobile phone-based surveillance system for rabies [45,46,47]; IBCM application [48]; ComD-STM [58]; SMS application for surveillance in humans and animals [62]; smartphone application for surveillance in humans and animals [62] and GoTHoMIS [63,64,65,66].
Disease detection	AfyaData app [43,44].
Disease reporting	eIDSR [10,33,34,35,36,37,38]; DHIS2 [38,39,40,41,42]; AfyaData app [43,44]; mobile phone-based surveillance system for rabies [45,46,47]; IBCM application [48]; MEEDS [49,50,51,52]; Coconut Surveillance [49,50,53,54,55,56,57]; SMS application for surveillance in humans and animals [62]; smartphone application for surveillance in humans and animals [62]; mobile application for collecting IDSR data [61] and ComD-STM [58].
Disease analysis	DHIS2 [38,39,40,41,42]; AfyaData app [43,44]; Coconut Surveillance [49,50,53,54,55,56,57]; ComD-STM [58] and GoTHoMIS [63,64,65,66].
Interactive feedback	AfyaData app [43,44].
Health facility-based surveillance	eIDSR [10,33,34,35,36,37,38]; DHIS2 [38,39,40,41,42]; MEEDS [49,50,51,52]; IBCM application [48]; GoTHoMIS [63,64,65,66]; smartphone-based reporting application [59,60]; mobile phone-based surveillance system for rabies [45,46,47] and mobile application for collecting IDSR data [61].
Community-based surveillance	AfyaData app [43,44] and Coconut Surveillance [49,50,53,54,55,56,57].
Mixed surveillance	ComD-STM [58]; SMS application for surveillance in humans and animals [62] and smartphone application for surveillance in humans and animals [62].
Single disease	MEEDS [49,50,51,52]; Coconut Surveillance [49,50,53,54,55,56,57]; mobile phone-based surveillance system for rabies [45,46,47]; IBCM application [48]; ComD-STM [58].
Multiple diseases	eIDSR [10,33,34,35,36,37,38]; DHIS2 [38,39,40,41,42]; AfyaData app [43,44]; mobile application for collecting IDSR data [61]; smartphone-based reporting application [59,60]; SMS application for surveillance in humans and animals [62]; smartphone application for surveillance in humans and animals [62] and GoTHoMIS [63,64,65,66].
Tanzania, mainland	eIDSR [10,33,34,35,36,37,38]; IBCM application [48]; ComD-STM [58]; smartphone-based reporting application [59,60]; mobile application for collecting IDSR data [61]; SMS application for surveillance in humans and animals [62]; smartphone application for surveillance in humans and animals [62] and GoTHoMIS [63,64,65,66].
Tanzania, Zanzibar	MEEDS [49,50,51,52] and Coconut Surveillance [49,50,53,54,55,56,57].
Nationwide	DHIS2 [38,39,40,41,42]; AfyaData app [43,44] and mobile phone-based surveillance system for rabies [45,46,47].

eIDSR: Electronic integrated disease surveillance and response system; DHIS2: District health management information system; MEED: Malaria epidemic early detection system; IBCM: Integrated bite case management; ComD-STM: Community-based disease surveillance and treatment of malaria system; SMS: short message service; GoTHoMIS: Government of Tanzania—hospital management information system.

**Table 5:** The purpose and benefits of mobile phones and computer-based systems identified from the literature review.

Reported Technology	Purpose	Benefits
eIDSR [10,33,34,35,36,37,38].	Reporting of weekly and immediately reportable diseases at health facilities.	Improved timeliness and completeness of reporting of above 80% national target. Linkage with the national health database provides timely data access to top authorities.
DHIS2 [38,39,40,41,42].	Reporting of weekly and monthly infectious diseases at health facilities.	Improved data availability and quality. Timely access and data sharing among stakeholders. Analysis of disease patterns and variations across districts/regions.
MEED [49,50,51,52].	Reporting of weekly malaria cases and notification of new malaria cases at the health facilities.	Within one year (2017–2018), the system increased weekly malaria reporting rates by 35% and 17% for Pemba and Unguja respectively. Enables detection and response to malaria outbreaks within two weeks of onset.
Coconut Surveillance [49,50,53,54,55,56,57].	Reporting of new malaria cases detected at the households by district malaria surveillance officers.	223 malaria cases detected through active surveillance at households in the first 6 months of rollout. Acknowledged to contribute to malaria elimination in Zanzibar.
AfyaData app [43,44].	Reporting of human and animal diseases in community settings by community health workers.	Around 1816 animal cases and 99 human cases reported during pilot. Assists with proper diagnosis and treatment. Assists timely epidemic detection in animals and humans.
Mobile phone-based surveillance system for rabies [45,46,47].	Reporting of animal bite cases presenting to health facilities to seek treatment.	Reported 29,595 cases of animal bites during pilot. Increased timeliness and completeness of the reports.
IBCM [48].	Reporting victims of animal bites seeking treatment at health facilities, and field reports of animal investigation by veterinary officers.	The number of reported bitten victims increased from an average of 55.7 to 92.2 following introduction of IBCM. Identification of rabies high-risk victims increased from 26.9–64.9% following introduction of IBCM. Assisted detection of 404 animals with rabies signs.
ComD-STM [58].	Reporting of malaria cases and treatment failure at community settings by community and health facilities.	Assisted detection of 1658 malaria cases and 9 malaria treatment failure cases during pilot phase.
Mobile application for collecting integrated disease surveillance and response data [61].	Reporting of weekly infectious diseases cases at the health facilities.	Improved timeliness of the reports from 50% to 89% during testing phase and saved time and cost of travel for submitting paper forms.
Smartphone-based reporting application [59,60].	Reporting of weekly integrated disease surveillance and response diseases and monthly reports at the health facilities.	Perceived by end users as quick and simple reporting tool compared to paper-based reporting. It is a low-cost intervention that prevents travel-related risk and gives more time to complete other tasks.
SMS application for surveillance in humans and animals [62].	Reporting of human and animal disease cases detected by community health reporters, reporting of animal cases detected by veterinary offices and reporting of weekly integrated disease surveillance and response diseases and monthly reports at health facilities.	No data on its implementation for surveillance activities but was used for data collection by PhD (Doctor of Philosophy) students.
Smartphone application for surveillance in humans and animals [62].	Reporting of human and animal disease cases detected by community health reporters, reporting of animal cases detected by veterinary offices and reporting of weekly integrated disease surveillance and response and monthly reports at health facilities.	Up to 1651 reporting forms submitted during pilot. Assisted writing of disease case reports and conducting follow-up.
GoTHoMIS [63,64,65,66].	Automated generation of weekly integrated disease surveillance and response disease reports at health facilities.	No evidence for its implementation in surveillance yet.

eIDSR: Electronic integrated disease surveillance and response system; DHIS2: District health management information system; MEED: Malaria epidemic early detection system; IBCM: Integrated bite case management; ComD-STM: Community-based disease surveillance and treatment of malaria system; SMS: short message service; GoTHoMIS: Government of Tanzania—hospital management information system.

## Data Availability

Not applicable.
